# Factors and clinical prediction score for complication development after cellulitis diagnosis in adult patients

**DOI:** 10.1186/s12245-024-00646-w

**Published:** 2024-05-22

**Authors:** Welawat Tienpratarn, Chaiyaporn Yuksen, Joseph Daniel Pauly, Diana Vu, Anisa Noiwong Benbourenane, Nuttamon Sangskul

**Affiliations:** 1 Department of Emergency Medicine, Faculty of Medicine Ramathibodi Hospital, Bangkok, Thailand; 2 Yale School of Medicine, Yale New Haven Hospital Emergency Department, New Haven, CT USA; 3 George Washington School of Medicine and Health Sciences, Washington, DC USA

**Keywords:** Prediction score, Cellulitis, Sepsis, Bacteremia, Necrotizing fasciitis

## Abstract

**Background:**

Cellulitis is defined as a bacterial infection of the skin and subcutaneous tissue that can cause multiple complications, such as sepsis and necrotizing fasciitis. In extreme cases, it may lead to multiorgan failure and death. We sought to analyze the clinical factors that contribute to the development of complicated disease, including demographics, clinical presentation, initial vital signs, and laboratory studies.

**Methods:**

Our study is a retrospective cohort study carried out in a university-based tertiary care hospital in Bangkok, Thailand. Adult patients who presented with cellulitis from January 1, 2018, to December 31, 2022, were evaluated for eligibility and inclusion in this study. All related variables for both outcomes, bacteremia and necrotizing fasciitis, were gathered from electronic medical records and analyzed using multivariable logistic regression analysis.

**Results:**

Of the 1,560 visits to this hospital, 47 cases reported at least one complication, with bacteremia noted in 27 visits (1.73%) and necrotizing fasciitis in 20 visits (1.27%). From the multivariable logistic regression analysis, six variables emerge as predictors of cellulitis complications. These are: Age ≥ 65 years, Body Mass Index ≥ 30 kg/m^2^, diabetes mellitus, body temperature ≥ 38 °C, systolic blood pressure ≤ 100 mmHg, and involvement of lower extremities. The predictive score was developed from these factors and was named the Ramathibodi Necrotizing Fasciitis/Bacteremia (RAMA-NFB) Prediction Score. Our predictive score has an accuracy of 82.93% (95% CI, 0.77–0.89). Patients in the high-risk group (RAMA NFB score > 6) have a likelihood ratio of 8.75 (95% CI, 4.41–18.12; *p* < 0.001) times to develop complications of cellulitis.

**Conclusion:**

In our study, the RAMA-NFB Prediction Score predicts complications of necrotizing fasciitis and bacteremia in adult patients who present with cellulitis. External validation of this predictive score is still needed for further practical application.

## Introduction

### Background

Cellulitis is defined as a bacterial infection of the skin or soft tissue, which can progress to bacteremia or necrotizing fasciitis if not treated. The incidence of cellulitis in patients with positive blood culture ranges from 4 to 30%, depending on the population and diagnostic criteria [1, 2]. The most prevalent bacteria cultured are Streptococcal and Staphylococcal species. These organisms account for 66–73.7% of all cellulitis-related bacteria. Moreover, Methicillin-Resistant Staphylococcus has been identified as a growing problem in hospital-acquired infections worldwide [2, 3].

The rate of hospitalization for skin infections varies depending on the severity of the disease and the patient’s comorbidities. Around 7% of patients with cellulitis are hospitalized, while mortalities range from 1 to 2.5%, depending on the study [3, 4]. Diabetes mellitus, chronic kidney disease, and peripheral arterial disease account for a higher rate of hospitalization and prolongation of hospital stays. One study from Siriraj Hospital in Thailand found that 20.6% of patients with cellulitis received inpatient care, with an overall mortality rate of 0.3% [3].

Apart from a patient’s comorbidities, several elements are regarded as risk factors for hospitalization for cellulitis, such as, increased age, immunodeficiency status, and area of skin involvement [5]. Swartz et al. suggest obtaining blood cultures in patients with cellulitis who have systemic symptoms (fever or chills), lymphedema with superimposed cellulitis, or tissue exposure to a non-sterilized body of water. They also suggest admitting patients who have failed outpatient management or those with rapidly spreading infection [2].

Proper care and early recognition of cellulitis are critical in preventing complications such as bacteremia or necrotizing fasciitis. Further studies are needed to evaluate patients at risk of developing limb or life-threatening complications from cellulitis. An appropriate scoring system may help identify high-risk patients and assist physicians in making clinical decisions on early intervention and management, both in the outpatient and emergency care settings. Additionally, the scoring system may guide hospitalization decisions, thus helping to reduce unnecessary health-care costs and decreasing length of stay times.

Some studies on clinical scores for cellulitis patients have been published. One study proposed the Melbourne ASSET (Area, Systemic features, Swelling, Eye, Tenderness) Score, is a tool to guide physicians regarding when to start intravenous antibiotics in children with cellulitis [5]. In terms of necrotizing fasciitis, Wong et al. proposed the LRINEC (Laboratory Risk Indicator for Necrotizing Fasciitis) score, which consists of laboratory items to identify the risk of developing early necrotizing fasciitis [6]. Another multi-center, prospective cohort study developed a risk score for predicting MRSA probability in adult patients with cellulitis [7]. Our study is the first to identify and provide a predictive score for complications in adult patients after a cellulitis diagnosis, in a university-based, tertiary care hospital setting without the need for laboratory investigations.

### Objectives

1. Explore factors associated with skin or subcutaneous soft tissue infections.
2. Create a scoring system to assist clinicians in identifying cellulitis cases that are high risk of developing complications of bacteremia or necrotizing fasciitis.

## Methods

### Study design and setting

We used a retrospective cohort, single-center model for this study. Our study was conducted at Ramathibodi Hospital in Bangkok, Thailand. Ramathibodi Hospital is a university-based, tertiary care center and an urban hub for training, education and referral services. The hospital serves a large patient volume, seeing a minimum of 5,600 outpatient visits daily and houses over 1,300 inpatient care beds. In 2023, the emergency department exceeded 42,000 annual patient visits. The hospital is a mega-tertiary care center with a wide range of specialties including dermatology, surgery, infectious disease, and burn services.

### Participants

Patients diagnosed with skin and soft tissue infection (coded as ICD-10 L03.9) during the study period were screened for eligibility. Only diagnoses listed as “new-diagnosis” or “new-episode” (in the case of recurrent cellulitis) during the study period were included. All follow up visits were excluded from the study. The inclusion criteria were: patients diagnosed with cellulitis, 18 years of age or older, and capable of follow-up for at least 1 month after the diagnosis of cellulitis [8, 9]. Patients from outpatient and emergency department visits were included in the study, with inpatient patient enrollment being excluded. Patients with incomplete data were excluded from this study.

### Duration of study

The study period was January 1, 2018 through December 31, 2022.

### Sample size

The sample size for our study was calculated based on a previous study by Lee et al. [10], which gathered data from patients with cellulitis with two different outcomes (positive blood culture group and negative blood culture group). The data from Tables 1 and 2 were used to calculate the sample size using Stata version 17.0 through a two-sample comparison of proportions and means. The assumptions were as follows: Alpha = 0.05 (one sided), Power = 0.8, and N2/N1 = 0.10. The smallest sample size that would produce a significantly different result was a total of 164 patients, comprising of 15 patients in the positive blood culture group (N1) and 149 patients in the negative blood culture group (N2).

### Data collection and study variables

From all medical records of cellulitis patients, 2,767 individuals presented with cellulitis within the 5-year study period, and 1,560 patients were eligible for our study based on inclusion criteria. The study variables were recorded for all eligible patients, including demographic data, characteristics of current cellulitis, past medical history, initial vital signs, and laboratory test results.

### Outcomes of interest

The outcomes of interest were the development of bacteremia or necrotizing fasciitis after an initial episode of cellulitis, and up to 1 month following the diagnosis. Bacteremia was defined as the presence of a positive blood culture result with cellulitis identified as the cause, according to the treating physician’s assessment. Necrotizing fasciitis was considered a complication when diagnosed in the patient’s medical records, whether by clinical or radiological diagnosis. Other complications associated with severe cellulitis, including septic shock, endocarditis, osteomyelitis or outcomes of ICU admission and mortality were not independently studied.

### Statistical analysis

Statistical analysis was conducted using STATA version 17.0 to create a prediction score. All eligible patients were categorized into two groups based on the presence of complications (complication and no complication group). Baseline characteristics were described using counts and percentages for categorical data, means and standard deviations for continuous data with a normal distribution, and medians and interquartile ranges (IQR) for continuous variables with a non-normal distribution. Data variables from both groups were compared using Welch’s t-tests and exact probability tests for continuous and categorical data, respectively. Using the rational clinical context, potential predictors that were significantly associated with complications were selected by multivariable logistic regression with backward elimination (p-value < 0.05). Regression coefficients for each level were divided by the smallest coefficient and rounded to produce the prediction score. The predictive power of the score was represented using the area under the ROC curve (both parametric and non-parametric) and a 95% confidence interval. Furthermore, the score-predicted risk and observed risk in our population were compared to demonstrate the predictive power of the score. Using this score, we categorized our patients into three groups: low-risk, moderate-risk, and high-risk. Positive likelihood ratios, 95% confidence intervals, and p-values were calculated for each group.

## Results

During the 5-year study period, 2,767 patient visits at Ramathibodi Hospital had a diagnosis of cellulitis. Among them, 1,560 (56.38%) patients were eligible for this study. Of these visits, 47 patients (3.01%) developed complications from cellulitis, including the outcomes of bacteremia (*n* = 27, 1.73%) and necrotizing fasciitis (*n* = 20, 1.28%).

Table 1 lists the clinical characteristics of the patients, sub-divided by the development of complications with a cellulitis diagnosis. In our study, complications were observed more frequently in males, with a total of 25 visits (53.19%). The mean ages in the complication and non-complication groups were 65.79 ± 15.78 years and 57.51 ± 18.97 years, respectively. Patients with complications from cellulitis had a significantly higher mean weight (75.53 ± 22.34 kg) and a higher mean BMI (29.56 ± 9.53 kg/m^2^) compared to the non-complication group. A higher incidence of diabetes mellitus was identified as a significant comorbidity in the complication group (55.32%) versus the non-complication group (20.16%). These two groups did not differ significantly with respect to prior wound, purulent features, or arterial lactate. However, cellulitis of the lower extremities tended to have significantly more complications (89.36% versus 53.28%). According to patients’ vital signs, those with complications from cellulitis had a significantly higher mean body temperature (37.56 ± 0.96 °C), respiratory rate (21.02 ± 3.56 breaths per minute), and lower mean oxygen saturation (96 ± 5.23%).

**Table 1:** Characteristics of patients with cellulitis. Results are categorized as complication or no complication after a diagnosis of cellulitis

Variables	Complication (N1 = 47)	No Complication (N2 = 1513)	*P*-value
Male	25 (53.19%)	574 (37.94%)	0.047
Age (mean ± SD)	65.79 ± 15.78	57.51 ± 18.97	< 0.001
Age ≥ 65 years	27 (57.45%)	594 (39.26%)	0.015
Weight (kg) (mean ± SD	75.53 ± 22.34	65.43 ± 17.82	0.004
Height (m) (mean ± SD)	1.60 ± 0.09	1.59 ± 0.10	0.332
Body mass index (kg/m^2^) (mean ± SD)	29.56 ± 9.53	25.60 ± 7.57	< 0.001
BMI ≥ 30 kg/m^2^	18 (40.00%)	260 (18.39%)	< 0.001
**Patient location**			< 0.001
Emergency Department	34 (72.34%)	241 (15.93%)	
Outpatient Department	13 (27.66%)	1272 (84.07%)	
**Underlying diseases**			
Diabetes mellitus	26(55.32%)	305 (20.16%)	< 0.001
Autoimmune disease	3 (6.38%)	58 (3.83%)	0.427
Malignancy	6 (12.77%)	157 (10.38%)	0.625
Cirrhosis	2 (4.26%)	17 (1.12%)	0.110
HIV infection	0	13 (0.86%)	1.000
Peripheral artery disease	0	24 (1.59%)	1.000
Chronic venous insufficiency	7 (14.89%)	87 (5.75%)	0.020
Lymphatic obstruction	2 (4.26%)	19 (1.26%)	0.130
Prior wound or infected wound	12 (25.53%)	223 (14.74%)	0.059
**Initial vital signs (mean ± SD), Number (%)**			
Body temperature (°C)	37.56 ± 0.96	36.92 ± 0.69	< 0.001
Body temperature ≥ 38 °C	15 (31.91%)	108 (7.14%)	< 0.001
Respiratory rate (breaths per minute)	21.02 ± 3.56	19.76 ± 1.53	0.020
Respiratory rate ≥ 22/min	10 (21.28%)	69 (4.70%)	< 0.001
Oxygen saturation (%)	96.00 ± 5.23	97.99 ± 1.56	0.034
Heart rate (beats per minute)	90.24 ± 19.05	84.94 ± 15.82	0.068
Heart rate > 90/min	23 (50%)	489 (32.95%)	0.025
Systolic blood pressure (mmHg)	129.26 ± 24.84	134.25 ± 21.80	0.179
Systolic blood pressure ≤ 100 mmHg	6 (12.77)	49 (3.30)	0.006
Decreased level of consciousness	1 (2.13%)	14 (0.93%)	0.369
Involvement of lower extremities	42(89.36%)	804 (53.28%)	< 0.001
Purulent	7 (14.89%)	13 (9.16%)	0.197
**Laboratory investigations**			
White blood cell count (median, IQR)	11,145 (5600–15,170)	8480 (6550–11,850)	0.101
Polymorphonuclear count (%) (mean ± SD)	77.20 ± 20.02	70.00 ± 14.46	0.021
Bicarbonate (mmol/L) (mean ± SD)	22.32 ± 3.40	23.53 ± 3.26	0.028
Creatinine (mg/dL) (median, IQR)	1.10 (0.77–1.62)	0.86 (0.69–1.18)	0.030
Venous Lactate (mmol/L) (median, IQR)	2.35 (1.60–3.50)	2.00 (1.50–6.20)	0.040
Arterial Lactate (mmol/L) (median, IQR)	2.77 (1.30–6.20)	2.40 (1.40–3.20)	0.656
Bacteremia	27 (6.77%) (total *N* = 399)		
Necrotizing fasciitis	20 (1.28%) (total *N* = 1560)		

**Fig. 1:**
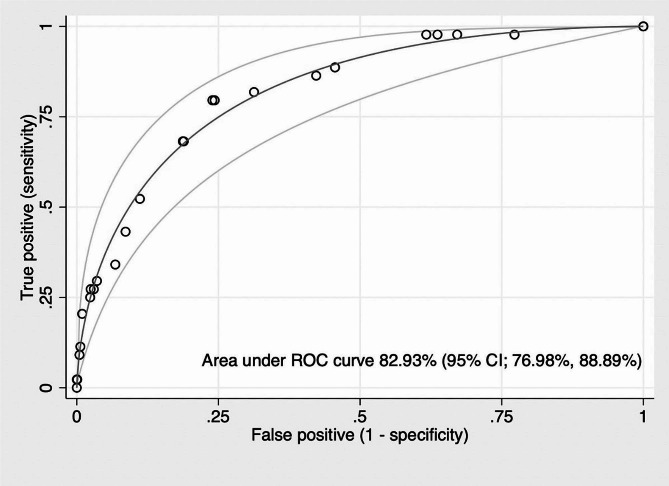
The Area under ROC curve (parametric) and 95% Confidence Interval for the predictive power of the RAMA-NFB Prediction Score for the development of cellulitis complications in adult patients

Multivariable logistic regression analysis was conducted to identify predictors of complication development following a diagnosis of cellulitis, as demonstrated in Table 2. The item score was determined by age (≥ 65 years),^12^ BMI (≥ 30 kg/m^2^) [11], the presence of diabetes mellitus, elevated body temperature (BT ≥ 38 °C) [12], low systolic blood pressure (SBP ≤ 100 mmHg), and cellulitis involvement of the lower extremities. The resulting prediction score was named the “Ramathibodi Necrotizing Fasciitis/Bacteremia (RAMA-NFB) Prediction Score.” The Area Under ROC of the clinical prediction score showed a predictive power of 82.93% (95% CI, 0.77–0.89) for complications after a cellulitis diagnosis (Fig. 1).

**Table 2:** Predictors of complication development and the assigned item score in cases of adult cellulitis (multivariable logistic regression analysis)

Predictors	Category	Adjusted Odds Ratio	95% CI	*P*-value	Coefficient	Score
Age ≥ 65 years	No Yes	1.00 1.57	Reference 0.82–3.05	- 0.186	- 0.45	0 1
Body Mass Index ≥ 30 kg/m^2^	No Yes	1.00 1.99	Reference 1.00-3.98	- 0.051	- 0.69	0 1.5
Diabetes mellitus	No Yes	1.00 2.82	Reference 1.43–5.55	- 0.003	- 1.03	0 2.5
Body temperature ≥ 38 °C	No Yes	1.00 5.03	Reference 2.50–10.10	- < 0.001	- 1.61	0 3.5
Systolic blood pressure ≤ 100 mmHg	No Yes	1.00 7.12	Reference 2.62–19.37	- < 0.001	- 1.96	0 4.5
Involvement of lower extremities	No Yes	1.00 4.05	Reference 1.52–10.81	- 0.005	- 1.40	0 3

**Fig. 2:**
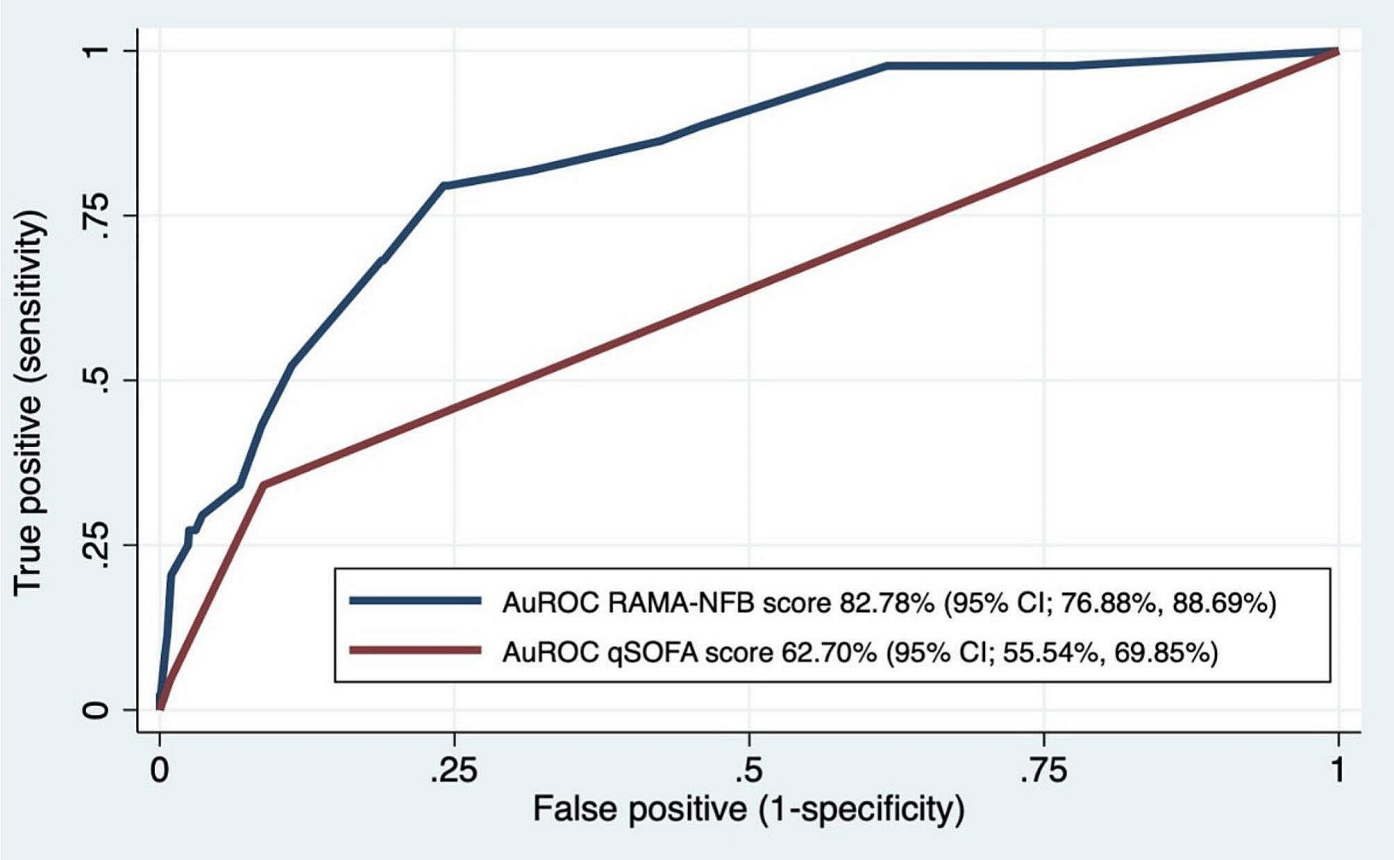
The Area under ROC curve (non-parametric) and 95% Confidence Interval of the predictive power of the RAMA-NFB Prediction Score for cellulitis complication development compared to qSOFA score

**Fig. 3:**
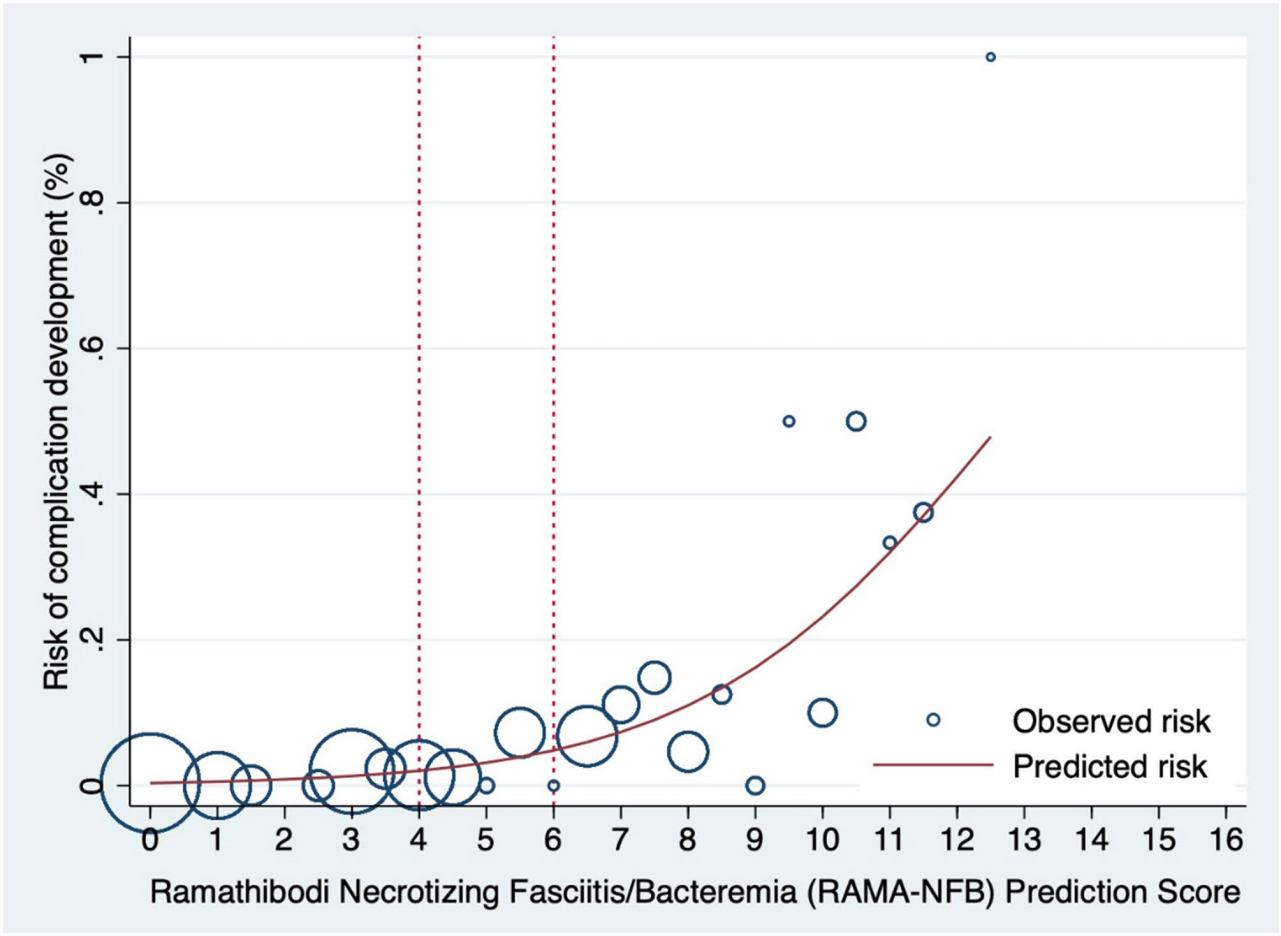
Observed risk (circles) versus score predicted risk (solid line) of complication development in adult patients with cellulitis

A comparison of the Area Under ROC by non-parametric analysis shows the predictive power of the RAMA-NFB Prediction Score to be 82.78% (95% CI, 76.88–88.69) compared to the qSOFA score of 62.70% (95% CI, 55.54–69.85) (Fig. 2). Additionally, the calibration of the prediction score is depicted, demonstrating the observed risk and predicted risk in adult patients with cellulitis (Fig. 3). Our clinical prediction scores were then categorized into three groups: low risk (score < 4), moderate risk (score 4–6), and high risk (score > 6). The probabilities of each score group are shown in Table 3.

**Table 3:** Probability categories in the RAMA-NFB Prediction Score for adult patients with cellulitis

Probability categories	Score	Complication (*N*, %)	No complication (*N*, %)	+ LHR	95% CI	Sens	Spec	PPV	NPV	*P*-value
Low	< 4	6, 13.64	731, 57.74	0.12	0.04–0.28	0.81%	93.4%	13.6%	42.3%	< 0.001
Moderate	4–6	8, 18.18	298, 23.54	0.72	0.29–1.60	2.61%	96.4%	18.2%	76.5%	0.473
High	> 6	30, 68.18	237, 18.72	8.75	4.41–18.12	11.2%	98.6%	68.2%	80.3%	< 0.001
Mean ± SD		7.11 ± 2.84	3.28 ± 2.71							< 0.001

**Fig. 4:**
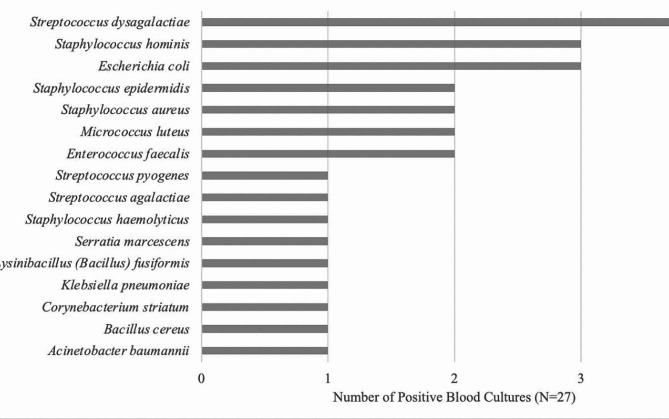
Bacterial species present in cellulitis-associated blood cultures

During their clinical evaluation, 399 patients had blood cultures collected. Of them, 27 (6.77%) were positive for bacterial growth (Fig. 4). 77.8% of the blood cultures grew gram positive bacteria with Staphylococcal species accounting for 29.6% and Streptococcal species accounting for 22.2% of the positive blood cultures. *S. hominis* (3 cases), *S. aureus* (2 cases) and *S. epidermidis* (2 cases) made up most of the staphylococcal blood stream infections. *S. dysgalactiae* was the most commonly grown organism (4 cases). 6 of the blood cultures grew gram negative bacteria (22.2%) with *E. Coli* being the most commonly grown gram negative organism, followed by *A. baumannii*, *K. pneumoniae*, and *S. marcescens.*

## Discussion

This study aimed to develop an initial prediction model that could assist physicians in determining the risk of complication development after diagnosing cellulitis in adult patients. The RAMA-NFB Prediction Score shows AuROC 82.93% (with 95% CI, 76.98–88.89). This indicates good correlation of the six identified variables (age ≥ 65 years, Body Mass Index ≥ 30 kg/m^2^, diabetes mellitus, elevated body temperature, low systolic blood pressure, and involvement of the lower extremities) to predict complications of bacteremia or necrotizing fasciitis following a diagnosis of cellulitis.

In previous studies [13, 14] cellulitis complications were more commonly observed in male patients. This was also identified in our study with a P-value of 0.047. Concerning underlying diseases, diabetes mellitus emerged as the most significant factor in this context. Our study’s outcomes align with those of Allen’s et al. previous study [15], showing the clinical impact of diabetes mellitus on a patient’s immune system. Surprisingly, comorbidities of autoimmune disease (N1 = 3, N2 = 58), malignancy (N1 = 6, N2 = 157), HIV (N1 = 0, N2 = 13), lymphatic obstruction (N1 = 2, N2 = 19) and peripheral artery disease (N1 = 0, N2 = 24) were not significant and did not emerge as independent predictors of complication development in patients with cellulitis. Interestingly, these findings may be related to close dermatologic monitoring or the use of prophylactic or early antibiotics in immunocompromised patient populations.

The study by Chamli et al. [16] showed that 94.9% of cellulitis cases occur in the lower extremities. The higher prevalence of lower extremity involvement in cellulitis from our study aligns with these findings. Furthermore, higher body weight and BMI contributed to the development of cellulitis complications, consistent with the study from Tianyi et al. [17] and Njim et al. [18] These findings highlight metabolic disease as a potential factor contributing to complication development from cellulitis.

Our study also demonstrates differences in findings compared with previous studies. Specifically, our study highlights the importance of patients’ initial vital signs as significant factors in the prediction of cellulitis complications. The cut-off point for temperature in our study, derived from Stevens et al. [12], was a temperature ≥ 38 °C, which emerged as significant vital sign predictor along with systolic blood pressure ≤ 100 mmHg. Despite several studies [19, 20] suggesting an elevated white blood cell (WBC) count, increased polymorphonuclear (PMN) cell count, or high serum lactate are indicative of more severe cellulitis, the clinical significance of laboratory investigations, in our study, are shown to have a lower impact. This allows the RAMA-NFB Prediction Score to be used early on in a patient’s diagnostic work up without requiring laboratory blood testing for implementation. Early use of the score may increase the speed of antibiotic interventions, disposition decisions, and in turn, reduce unnecessary hospitalizations and health-care costs. Additionally, this clinically based decision-making tool may be useful in low-resource settings where laboratory investigations are not readily available.

Our findings demonstrate that by considering a combination of a patient’s age, BMI, underlying diseases, vital signs, and the location of cellulitis, it is possible to predict the likelihood of complications arising from cellulitis. This information can be valuable in assisting physicians with their clinical decision-making. We suggest that if patients were categorized into a low-risk group (score < 4), the probability of cellulitis developing into bacteremia or necrotizing fasciitis would be low with a positive likelihood ratio 0.12 (95% CI, 0.04–0.28; *p* < 0.001). Thus, management for this group of patients can be limited to an outpatient setting. Conversely, in the high-risk group (score > 6) of patients, there is a high probability of complications developing after cellulitis diagnosis with positive likelihood ratio 8.75 (95% CI, 4.41–18.12; *p* < 0.001). We would hypothesize that the high-risk group might benefit from more aggressive management, including obtaining blood cultures, admission for close monitoring, and administration of early antibiotics. For patients identified as moderate risk (score 4 to 6) for developing complications, further discussion should be provided to the patient and their relatives regarding the risks and benefits of the treatment plan, and patients should be followed up to detect any potential complications.

### Limitations

Our center is a high-volume, urban, university-based, tertiary care hospital; thus, patients are more likely to have complicated underlying diseases or a higher risk of exposure to advanced organisms compared to those in community-based rural hospitals. Because this study is conducted at a single center, there may be limitations in generalizability to other populations or demographics. Therefore, further external validation of our predictive score is needed using independently obtained patient data of other hospital systems to validate the model. Additionally, the absence of randomization and use of a retrospective study makes controlling for confounding variables and establishing a causal relationship of the identified outcomes difficult. To improve generalizability and causality, a multi-center prospective investigation could be pursued.

Furthermore, our study focused on the development of two specific complications: necrotizing fasciitis and bacteremia. Additional studies to explore outcomes of septic shock, septic arthritis, endocarditis, osteomyelitis, mortality, and other cellulitis-associated complications would be useful to broaden applicability of the score.

Notably, the use of ICD coding for enrollment creates a standardized, dichotomous system for enrollment, however, limitations exist when characterizing purulence or stratifying the extent of soft tissue involvement. Further studies to better understand varying degrees of cellulitis as a contributing factor for complication development would be useful in aiding in clinical decision making for admission.

Lastly, recent antibiotic use was not included as a variable in this study. Use of antibiotics prior to enrollment could confound the development of necrotizing fasciitis, bacteremia, or other associated complications of cellulitis in the enrolled patient population. As a result, the RAMA-NFB score may have limitations when applied to patients taking antibiotics. Further research to evaluate recent antibiotic use as an independent predictor should be investigated.

## Conclusion

In conclusion, the RAMA-NFB Prediction Score can help predict the risk of complication development for adult patients with cellulitis. The high-risk patient group (with a score > 6) is more likely to progress to complications, either bacteremia or necrotizing fasciitis. The RAMA-NFB score allows for early decision making in diagnosis, disposition, and treatment. Furthermore, the clinical score does not require laboratory testing, thus making implementation easy to use and applicable to Emergency Departments with varying degrees of resources.

## Data Availability

No datasets were generated or analysed during the current study.
